# Implementing internet-delivered cognitive behavioral therapy in healthcare services: a qualitative exploration of stakeholder experience

**DOI:** 10.3389/fdgth.2023.1139125

**Published:** 2023-09-26

**Authors:** Daniel Duffy, Derek Richards, Caroline Earley, Ladislav Timulak

**Affiliations:** ^1^ SilverCloud Science, SilverCloud Health, Dublin, Ireland; ^2^ School of Psychology, Trinity College Dublin, Dublin, Ireland

**Keywords:** iCBT, digital interventions, implementation research, qualitative research, depression, anxiety

## Abstract

**Background:**

This study consisted of a qualitative exploration of stakeholder experience regarding the implementation of internet-delivered cognitive behavioral therapy (iCBT) as part of routine service provision within the UK's Improving Access to Psychological Therapies program.

**Methods:**

Stakeholder groups included service providers (*n* = 6), commercial iCBT representatives (*n* = 6) and patients who received a course of iCBT as part of treatment at the service (*n* = 7). Participants took part in a semi-structured interview over the telephone, and subsequent data were qualitatively analyzed using the descriptive-interpretive approach.

**Results:**

Service providers highlighted the importance of effective leadership and management, training initiatives, the provision of feedback to commercial iCBT representatives and creating work structures around iCBT to facilitate iCBT supporting staff in their use of it. Commercial iCBT representatives similarly reported the importance of training clinicians in iCBT use, identifying the appropriate individuals at all levels of the service to drive iCBT implementation, and the importance of being responsive to any problems or needs that arise from the service. Patients reported an overall positive experience of receiving iCBT but highlighted a need for more information from their supporter and the intervention to better structure their usage. Contextual factors, in terms of barriers and facilitators, were also highlighted by service provider and commercial participants; citing negative clinician attitudes and technological issues/bugs as barriers to implementation, and the exposure to iCBT created by COVID-19 and perseverance in using iCBT over time as facilitators.

**Discussion:**

The findings contribute to a growing field of literature that seeks to understand the experience of relevant stakeholders who are involved with and contribute to iCBT implementation, including commercial iCBT representatives who, to our knowledge, have not been accounted for as part of published research to date.

## 1. Introduction

Internet-delivered cognitive behavioral therapy (iCBT) interventions are a novel and convenient means of providing effective mental healthcare and are stated to overcome the barriers associated with traditional modalities of psychological treatments (1, 2). iCBT has illustrated its effectiveness and efficacy across numerous trials (3–6) but its adoption as part of routine care provision has been limited (7). This is illustrative of an “evidence-to-practice” gap (8–10), where evidence-based interventions (like iCBT) are underused, misused, or perceived by professionals to be inferior to current practice (10). Implementation Science (hereafter IS) is the study of methodologies and approaches associated with understanding and increasing the uptake of novel, evidence-based practices within healthcare (11, 12). Within the field of iCBT, it has been cited that adopting methods from the field of IS can help facilitate the uptake of iCBT within routine care (7, 13–15).

The field of iCBT is growing quickly, but the availability of evidence that reports on relevant stakeholder experience of implementation and associated factors is lagging. Accordingly, our understanding of ‘iCBT in the wild’ is limited, and this problem is compounded by a dynamic healthcare context that is constantly evolving. Some qualitative explorations of the use of iCBT within services exist (13, 15, 16), and even fewer have simultaneously taken account of three stakeholder groups that feature heavily within the lived reality of iCBT; commercial iCBT representatives, service providers who routinely use it as part of their practice, and patients receiving iCBT (15). Of note, the impact of commercial iCBT representatives on the implementation of iCBT is often neglected or unmentioned. However, it is understood that services perceive positively the support they receive from “external facilitation units” (15), that provide support to services implementing iCBT and may fulfil a role similar in function to commercial iCBT company representatives.

Regarding commercial iCBT representatives, numerous companies have come into existence that have grown around a currently booming digital healthcare market (17). These entities have vested commercial interest in ensuring that their products deliver on promised clinical outcomes, and generally work quite closely with healthcare services to foster successful implementations. Ignoring the impact of commercial entities, the teams behind them and their role in the implementation process and consequent outcomes from iCBT is no longer possible; they bring a wealth of expertise when it comes to successful implementation. Similarly, we know that patients experience iCBT positively (3, 18, 19), but the patient experience in regards to the procedures they encounter during their treatment experience of iCBT is still not sufficiently explored. Patients are the ultimate ‘receivers’ of iCBT, who reap the benefit or pay the cost of an implementation effort, and therefore capturing the experience of multiple stakeholders involved with implementation initiatives is important for the field going forward.

## 2. Methods

### 2.1. Design

Utilizing a descriptive-interpretive approach (20), the current study qualitatively analyzed stakeholders’ experience of implementing iCBT in mental healthcare services. The current study departs from existing research by including commercial iCBT representatives, service providers and patients to examine more holistically the phenomenon of implementing iCBT in a mental healthcare service. No specific implementation theory, model or framework (TMF) was chosen to guide the design of this study for several reasons. Firstly, the goal of this study was to generate a bottom-up understanding of implementation experiences within a routine services across the three participant groups; no a-priori rationale was identified to use a specific TMF. Second, and related to the first point, no member of the author group was aligned to one specific TMF or could identify a rationale to use one TMF over another. The research sought to capture the roles of different key stakeholders in implementing iCBT, context in which it occurred and the implementation factors of most importance. We therefore settled on an inductive approach, where two domains of interest were developed: experience of iCBT implementation and contextual considerations for implementing iCBT.

### 2.2. Participants

Between June-October 2020, 19 participants were recruited. Service providers (*n* = 6) were invited to participate in the study through managers within the service, and participants in the service provider group occupied roles such as Psychological Wellbeing Practitioner (PWP; low intensity therapist, hereafter referred to as “supporters”) (*n* = 3), innovation-pathway lead (*n* = 1), administrative manager (*n* = 1) and service director (*n* = 1). Participants in this group were employed by an Improving Access to Psychological Therapies (IAPT) service, which is a service that provides mental health care within primary care. Participants were required to have first-hand experience of working with the intervention, as well as participating in implementation initiatives such as training, product improvement and personnel management. Importantly, the NHS organization employing participants in the service provider group would be classified as “high-performing”, in that it meets or exceeds NHS standards for recovery and symptom improvement rates across its service offerings. Further, the NHS organization has significant experience of using iCBT, having employed numerous commercial platforms since 2012 as part of their routine care services and employs approximately 60–70 PWPs at the low-intensity level to deliver these interventions to a catchment area of 900,000 people.

Commercial iCBT representatives (*n* = 6) were recruited similarly (e-mails from managers within the commercial entity), and included job roles such as customer success (*n* = 1), sales (*n* = 1), marketing (*n* = 1), product (*n* = 2) and software (*n* = 1) development. Service providers recruited patient participants at their final treatment session prior to iCBT completion. Patient Participants (*n* = 7) had completed a minimum of 4 sessions of iCBT as part of their step 2, low-intensity treatment within an NHS primary care mental health service (IAPT). Participants in the patient and service provider stakeholder groups were provided with a £10 online shopping voucher to reimburse them for contribution. For an overview of participants included in the study see Tables 1, 2.

**Table 1:** Characteristics of participants in the commercial iCBT representative and service provider groups.

Participant ID	Age	Gender	Group	Role	Years in role
1	38	m	Commercial iCBT Representative	Sales	2
2	35	m	Commercial iCBT Representative	Customer success	2
3	42	f	Commercial iCBT Representative	Product	10
4	42	f	Commercial iCBT Representative	Marketing	6
5	40	m	Commercial iCBT Representative	IT Developer	11
6	48	m	Commercial iCBT Representative	Product	2^a^
7	32	f	Service Provider	PWP/Digital lead	3
8	28	f	Service Provider	PWP/Digital lead	2.5
9	31	f	Service Provider	PWP/Service lead	2^b^
10	38	f	Service Provider	Manager – service lead	3^c^
11	36	f	Service Provider	Manager – administrative	1
12	60	f	Service Provider	Director of service	8

^a^ Participant stated they had substantial experience of working within NHS services as management staff before commencing this role.

^b^ Participant had worked within the service for 6 years as a Psychological Wellbeing Practitioner and team lead before commencing this role.

^c^ Participant had had worked within the service for 9 years as a Psychological Wellbeing Practitioner and manager before commencing this role.

**Table 2:** Characteristics of participants in the patient group.

Participant ID	Age	Gender	Referral origin	Support received	Status^a^
13	25	f	GP advised patient to self-refer	Telephone and online summaries	Discharged
14	43	f	GP advised patient to self-refer	Telephone and online summaries	Waiting list for further therapy
15	40	f	GP referral	Telephone only	Completing treatment
16	25	m	Self-referral	Telephone only	Discharged
17	49	m	GP referral	Telephone and online summaries	Discharged
18	28	f	Self-referral	Telephone and online summaries	Waiting list for further therapy
19	64	f	GP referral	Telephone and online summaries	Discharged

^a^ At the time of the interview being conducted.

### 2.3. Semi-structured interview – overview

A semi-structured interview focussing on two domains of investigation was developed for the commercial iCBT representatives, service providers and patients. Specifically, these domains were *experience of iCBT implementation* and *contextual considerations for implementing iCBT*. To this extent, a literature review of relevant theories, models and frameworks (TMFs; e.g., Birken et al., 2018) in the field of implementation science was conducted. However, it was then decided that, instead of using one specific TMF to guide the interview schedule, the interview would take a broader, inductive approach to stakeholder experience. As part of *experience of iCBT implementation*, the research team separated this domain into two sub-domains that were 1) Implementation process, with questions that explored participant's experience of what they *do* as part of implementation of iCBT [e.g., implementation strategies; (Powell et al., 2015; Proctor et al., 2013) and 2] Decisive elements for successful implementation, which related to the factors that are *most important* to each of the relevant stakeholders regarding implementation. The domain *contextual considerations for implementing iCBT* was concerned with the factors in the immediate or wider context that may potentially impact on iCBT, its usage and implementation. A copy of the interview schedules for each participant group are included as part of the Supplementary Material.

### 2.4. Ethics

Human subjects’ approval was provided by the School of Psychology, Trinity College Dublin for participants residing in Ireland, and by the Health Research Authority of the United Kingdom (IRAS ID: 270142). All participants were provided with information sheets that detailed study procedures and data processing information and were then required to sign informed consent prior to participating.

### 2.5. Researcher characteristics & reflexivity

The first author, DD, has worked with SilverCloud Health as a researcher within the field of online interventions for a period of 8 years, mainly involved with research trials within England's National Health Service, training clinical staff and services in the use of the platform and content development for several iCBT programmes. CE has similarly worked with SilverCloud as a researcher and project manager for 7 years, and manages the delivery of the Science team's portfolio of research. DR is Chief Science Officer at SilverCloud Health and has worked in the internet intervention space for over 15 years. LT is a counselling psychologist, with an expertise in qualitative methodologies and interest in client experiences of psychotherapy. Both DR and LT have extensive research and career experience in the field of internet interventions and provision of psychological therapies.

### 2.6. SilverCloud Health

The company that develops the SilverCloud iCBT intervention is SilverCloud Health, and has been operating within the field of digital therapies since 2012. Specifically for the IAPT market, SilverCloud is used in a large number of services at the Step 2 (low-intensity) level to treat common mental health disorders (mainly depression and the anxieties). In the current study, the interventions used by patients and administered by staff consist of ‘*space from depression*’ and ‘*space from anxiety’* (or anxiety delineation, e.g., generalized anxiety disorder, social anxiety). The interventions all share core CBT components (e.g., psychoeducation, tools including a mood monitor, thought-feeling-behavior cycles, cognitive restructuring) with tailoring (e.g., differing clinical vignettes for content illustration purposes) for the target conditions. SilverCloud can be delivered in self-guided (unsupported) or supported formats, with support delivered either synchronously (over the telephone, video call) or asynchronously (using an in-built messaging feature within the app or web experience). A full list of references utilizing SilverCloud interventions across multiple contexts of care and geographies can be found on the company website (https://www.silvercloudhealth.com/our-research).

### 2.7. Data analysis

The qualitative data were analyzed using the descriptive-interpretive qualitative method of Elliott & Timulak (20). At a base level, the descriptive-interpretive approach involves breaking the data into ‘meaning units’, consisting of qualitative data that convey meaning to a reader even when taken out of context. These meaning units are then assigned to the domains of investigation, clustered together according to their similarities, and are subsequently categorized to produce insights and the ultimate findings. As part of this analytic process, new domains of investigation or sub-domains can occur that may differ from or extend the original domains of investigation. Specifically, the analysis adhered to the following steps

(1) The qualitative interview recordings and transcripts were reviewed numerous times by DD to become familiar with the dataset and what participants reported.
(2) Interview transcripts were then analyzed by DD, where the data were broken down into discrete meaning units and summary labels were created for these that conveyed a shorthand explanation of what was reported by participants.
(3) Once the final data set of the meaning units was established by DD, they were extracted from their original transcripts to a spreadsheet (Microsoft excel) to allow for accurate indexing and analysis. Data were then organized according to the pre-existing domains of investigation; Experiences of iCBT Implementation and contextual considerations for implementing iCBT*.* This allowed for a preliminary structure to be established within the dataset, but domains were not finalized until the categorization process was completed.
(4) The meaning units within the domains of investigation were then reviewed and clustered according to similarities, which began the process of categorization and was carried out by DD. The process of categorization within the descriptive-interpretive method is subjective and interactive; the categorization of the data corresponds to and is impacted by both the meaning units illustrated by participants and the background interpretive framework of the researchers conducting the analysis. This process also highlighted the presence of relevant sub-domains.
(5) Category names were continuously revised to ensure they best represented the data, which further allowed for the meaning units within each category to be interrogated for fit. Any amendments proposed to categories throughout this process were discussed across the research group and, where appropriate, were actioned.
(6) The final domain structure was established once categorization was completed. While domains organize the presentation of findings, categories summarize and contain the actual findings.
(7) Auditing: Throughout the analysis of transcripts, DD, DR and LT met weekly to audit the ongoing process. Where there was a lack of clarity around certain meaning units, domain allocation or the generation of categories (e.g. splitting of a single meaning unit into multiple meaning units), DD would present this and seek consensus.

## 3. Results

Qualitative data are presented across the 3 participant groups below. The first domain of interest, experience of iCBT implementation, yielded three separate domains of analysis for each of the participant groups, and each domain of analysis is presented separately for the three groups. This consisted of ‘commercial iCBT representative implementation strategies’ (domain 1) for commercial iCBT representatives (*n* = 6), ‘service provider implementation strategies’ (domain 2) for service providers (*n* = 6) and ‘patient experience of an iCBT treatment pathway’ (domain 3) for patients in the service (*n* = 7), across five sub domains. Contextual considerations for the implementation of iCBT (domain 4) provides findings from commercial iCBT representatives and service providers, divided into two sub-domains: ‘contextual barriers’ and ‘contextual facilitators’. Data associated with the sub-domains under contextual considerations are presented in combined format, with data from both commercial iCBT representatives and service providers presented (*n* = 12). Tables 3–6 present the domain and category structures associated with the aforementioned domains. Table 7 presents a breakdown of participant contributions to each of the categories in the domain “Contextual Considerations”.

**Table 3:** Categories associated with domain 1 – commercial iCBT representative Implementation strategies, based on data from the commercial iCBT representative group (*N* = 6).

Category	*N*
Being responsive to service provider needs to provide guidance and troubleshoot issues	6
Identifying “the right people” within services at all levels (directorial, managerial, frontline worker)to implement, sustain and develop iCBT in mental health services	5
The training of supporters and coaches in the use of iCBT by the intervention developer to be proficient in its administration	4
Building the required team structure to ensure successful implementation and scaling of iCBT	4
Working with the service provider to integrate iCBT within care pathways	4
Conducting product pilots with services to demonstrate use cases for new programs	3
Working with more services negatively impacts on the availability of resources to support multiple, concurrent implementations of iCBT	3
The development of online resources, including webinars and online training courses	2
Educating potential referrers in iCBT	1

**Table 4:** Categories and sub-categories associated with domain 2 – service provider Implementation strategies, based on data from the service provider group (*N* = 6).

Category	Sub-category	*N*
Implementing and enacting effective leadership systems to support the use of the intervention and assist supporters in its utilisation	The importance of having management with capacity to drive change and accommodate the delivery of digital as part of service provision	6
	The role of digital champions in pioneering iCBT within the service to allow it reach its full potential	6
	Visibility and clarity of goals related to iCBT, and their role in overall service provision	3
In-service training initiatives to educate supporters in the use and benefits of iCBT	On-going training to highlight new programs or procedures related to iCBT is important	6
	Training initiatives for new starters (trainees and recently hired clinicians) in the use of iCBT within the service are necessary to build supporter competency	5
	Disseminating clinical outcomes of iCBT to demonstrate effectiveness and encourage use among staff	5
Conveying feedback to intervention developers is important in improving the iCBT offering and maintaining a good commercial relationship	Positive perceptions of service providers on their relationship with the iCBT company creates feelings of ‘working in partnership’	4
	Gathering feedback on iCBT programs and their content is important in addressing the needs of clients	4
	Gathering feedback on gaps in iCBT service provision improves its use among staffs within services	3
Creating iCBT appropriate work structures facilitates supporters in its delivery	Creating tools and reference documents supports staffs in their use of the intervention	5
	Line management is important in establishing and monitoring individual staff goals around iCBT use that are reflective of wider service goals	5
	Designing and revising existing pathways for iCBT use facilitates its performance in terms of clinical outcomes and access	4
	Routinely auditing iCBT data is important in improving how the service administers iCBT	3
	Clinical supervision is valued in supporting iCBT provision and helps to address issues of clinical risk	2

**Table 5:** Sub-domains and categories associated with domain 3 – patient experience of an iCBT treatment pathway, based on data from the patient group (*N* = 7).

Sub-domain	Category	*N*
Patient experience of the iCBT platform	Patients state the flexibility and accessibility of the platform as positive aspects of iCBT	6
	Patients appreciated that the program contained appropriate content and tools to address the problems the person is going through	5
	Patients appreciated how the platform enabled them to take and use the content they needed, while filtering out content that was less relevant	4
	Patients expressed a need for more guidance within the intervention regarding how to effectively use it	4
	Issues with platform functionality, including tool layouts, presentation of questionnaires, length of mindfulness exercises and security features (i.e. requiring repeated logins)	4
	The integrated reminder function on the platform is helpful and useful in structuring patient iCBT usage	3
	Patients who had received previous therapy (e.g. face-to-face CBT) reported that iCBT and its content was not redundant	3
	Patients appreciate being able to download and print content for instances with no internet connection	2
	Patients reported the platform to be an aesthetically pleasing experience	2
Patient experiences of the administration of treatment by the service	Positive assessment experience; supporters collaborated with patients to decide on iCBT and normalised their treatment-seeking.	6
	Feeling supported by supporter to prepare for discharge from iCBT.	5
	Clear and defined procedures for cancelling or rescheduling treatment appointments	3
	Multiple reminders (text message and e-mail) sent by the service helped to maintain engagement in treatment	2
Patient experiences of their clinical supporter	Patients found typed summaries of telephone calls using the online support function helpful in structuring their future use of the program	5
	Patients reported that more guidance is needed from the service regarding how to use the program and its tools	4
	Patients appreciated when supporters tailored content recommendations based on their presenting problems	3
	Patients stress the importance of telephone supporter support in increasing adherence and normalising presenting problems	2
	Patients reported that the initial awkwardness of telephone supported was alleviated by the supporter's skill	1
Patient experience of the service referral process	Patients reported positive experiences of the online, self-referral process	3
	Patients reported speaking with GPs regarding mental healthcare as an easy and positive experience	3
	Patients report a preference for online referral over healthcare provider referral when they have previous negative experiences with treatment seeking	1

**Table 6:** Sub-domains and categories associated with domain 4 – contextual considerations for the Implementation of iCBT, based on data from the service provider and commercial iCBT representative group (*N* = 12).

Sub-domain	Category	*N*
Contextual Barriers	Negative clinician attitudes towards ICBT can limit opportunities for implementation	**8**
	Technological issues associated with iCBT's interoperability with patient management systems can be a barrier	**5**
	Market variability may negatively impact on the resources needed to implement iCBT	**4**
	The rigid requirements of care pathways may limit the application of iCBT, similarly in-service bureaucracy when trying to further iCBT	**3**
	Services need to train new hires in iCBT due to PWP training programs not covering it in sufficient detail, creating false expectations of the role and therapeutic work involved.	**3**
	Costings & Pricing Models as a barrier to implementation	**3**
Contextual Facilitators	COVID-19; changing the way service is delivered due to cessation of face-to-face services, resulting in greater exposure of clinical staff to iCBT	**10**
	The passage of time and perseverance in using iCBT facilitates implementation by allowing for services to understand and improve their iCBT offering	**8**
	Support for the use of digital technologies within the wider health system is facilitative of iCBT adoption and implementation.	**6**
	Organizational culture within mental health services can facilitate iCBT implementation	**4**
	Periods of staff shortages may create increased reliance on iCBT usage	**2**

**Table 7:** Commercial iCBT representative and service provider individual contributions to sub-domains associated with “contextual considerations for the implementation of iCBT”, where numbers 1–6 are commercial iCBT representatives and 7–12 are service providers.

Sub-domain	Category	*N*	Participant no.											
			1	2	3	4	5	6	7	8	9	10	11	12
Contextual Barriers	Negative clinician attitudes towards ICBT can limit opportunities for implementation	8			x	x	x	x	x	x	x	x		
	Technological issues associated with iCBT's interoperability with patient management systems can be a barrier	5		x			x				x	x		x
	Market variability may negatively impact on the resources needed to implement iCBT	4	x	x	x		x							
	The rigid requirements of care pathways may limit the application of iCBT, similarly in-service bureaucracy when trying to further iCBT	3		x		x								x
	Services need to train new hires in iCBT due PWP training programmes not covering it in sufficient detail, creating false expectations of the role and work involved	3							x			x		x
	Costings & Pricing Models as a barrier to implementation	3	x	x										x
Contextual Facilitators	COVID-19; changing the way service is delivered due to cessation of face-to-face services, resulting in greater exposure of clinical staff to iCBT	10	x	x	x	x		x	x	x		x	x	x
	The passage of time and perseverance in using iCBT facilitates implementation by allowing for services to understand and improve their iCBT offering	8			x	x	x	x	x	x	x	x		
	Support for the use of digital technologies within the wider health system is facilitative of iCBT adoption and implementation.	6	x		x	x	x		x		x			
	Organisational culture within mental health services can facilitate iCBT implementation	4							x			x	x	x
	Periods of staff shortages may create increased reliance on iCBT usage	2										x		x

For commercial iCBT representatives and service providers, it was found that when asked to identify “decisive elements” within the process of implementation, they reiterated or explored further previous statements they had made. For patients, it was found, even with the provision of prompts, that they were unable to comment thoroughly on the impact of context on their use of iCBT. Given these findings, the domains of “decisive elements” for commercial iCBT representatives and service providers, and “context” for patients are not reported on.

### 3.1. Domain 1: commercial iCBT representative implementation strategies

According to commercial iCBT representatives, training provided by commercial iCBT representatives conveys how the program works, its benefit to clinical services (who are customers of the iCBT company), how to make decisions around suitability for iCBT, teaches supporters the basics of online therapeutic communication and also how to develop scripts to inform prospective patients about iCBT. Practical sessions (*“click-throughs”)* are also conducted to teach supporters how to navigate the online platform and set their clients up with iCBT accounts. The length of training can span from a 2-hour session to an entire day. Training could also be delivered to referring health professionals, such as GPs and indeed the provision of online resources, such as research papers on iCBT evidence base and case studies from other customers. Commercial iCBT representatives conduct a number of product piloting initiatives with interested customers to develop use cases for new iCBT programs. As part of these pilots, program specific educational materials (training presentations, guides) are developed and provided to customers by commercial iCBT representatives, clinical outcome data is collected to judge the effectiveness of the program and pathways are worked on to fit the intervention into the current care offering. Results of these pilots then contribute to case studies.

Building and organizing the correct team structure to carry out implementation activities is important. Developing a multi-disciplinary organization, consisting of marketing, sales, design, clinical, research and commercial departments, has allowed for the creative problem solving of implementation problems. This team provides guidance to customers throughout the implementation process regarding care pathway set-up and integration. One participant stated “*… having the right people at the table … definitely [supports] real-world [delivery] and results”*. At a service level, the “right people” influence decisions (e.g., service managers, leaders, directors), or are responsible for the direct administration of the intervention, as “*they’re the ones who can make a difference*”. Digital champions, described by participants as advocates for iCBT, are important at all levels (e.g., from clinical lead to supporters) as not only do they drive the use of the intervention within day-to-day service workings, but can also become peer leaders who “*…can bring along the rest of their teammates with them”.* Further, top-down only implementation, where procedures implemented may not fit well with routine care practices are complimented by bottom-up approaches, which promote intervention utilization via peer-influencers within the organization.

Understanding the customer use case is highly important for both commercial viability and creating value for customers. One participant stated that this is done through a process of “*talk[ing] to customers in the language of the jobs they need doing”*, which creates a focus on their goals for iCBT. Sometimes customers are unable to identify efficiently “*what is a proper problem”*. Commercial iCBT representatives highlighted that they have many years of experience allows them to make recommendations to customers when they face barriers to implementation.

Commercial success and the need for concurrent implementations can subsequently deplete the amount of available human resource. This can make customers feel like they are not receiving enough attention as they try to implement iCBT. Due to this lack of resources, participants stated that they are unable to examine certain customer aspects (e.g., iCBT license usage, need for training materials, general service reviews) that would typically facilitate growth in the service providers’ use of iCBT.

### 3.2. Domain 2: service provider implementation strategies

Participants emphasized the importance of having effective management and a culture of passion for the delivery of digital therapies. One participant stated that “*I think SilverCloud wouldn't have been successful if we hadn't had people at the top of the service– the directors, the senior leadership team, who also really invested in iCBT as well”*. Having a senior implementation driver within the organization who is “*strategic…and forward thinking*” in regard to digital intervention usage within the service ensures the continuation of the implementation effort. Goals often mandated by the national health authority, are mainly set around “*make[ing] sure we [the service] increase the use of this [iCBT] program”*, and senior management work across therapeutic departments (e.g., low-intensity therapy, high-intensity therapy) to ensure that learnings from one use-case are scaled to other therapy areas. Digital champions, as described by participants, are staff members (including clinical and administrative roles) within service teams that are passionate about iCBT and digital ways of working. They trial new digital initiatives within their teams (e.g., new iCBT programs), train other staff members in digital, lead on research projects, collect feedback within their teams, respond to client queries, and problem solve barriers to digital uptake using a data informed approach.

Comprehensive training programs for new starters (including trainees and recently hired supporters) was highlighted by service providers as necessary. Training includes familiarization with the different iCBT programs, observing another supporter in their use of iCBT, being observed in their use of iCBT and roleplaying as a client. Supporters also observe others and are observed during the assessment/triage process to ensure appropriate treatments (e.g., iCBT, bibliotherapy) are assigned to patients. Online training materials support the effort, and are used to supplement in-person efforts. On-going training is important for senior managers, supporters and administrative staff. Digital champions were cited to be important for training staff, as they “*do that kind of deep-dive, understanding it, and then share that learning”*. Some staff receive targeted training “*to support them, to understand the barriers to why they’re not using it and to support them to overcome some of those barriers”*. Disseminating iCBT outcome data creates buy-in from supporters and other clinical staff. Outcome data based on the performance of iCBT in terms of the recovery rates of patients across different programs is sent to all staff monthly. The data “*speaks for itself”* and helps to show that the treatment is evidence based and can achieve valid clinical outcomes.

Feedback on iCBT service provision is stated to largely come from the supporters who are using iCBT as part of routine service. It normally occurs via new iCBT initiatives but can also relate to barriers experienced by supporters and is not limited to iCBT. Issues with the content and platform functionality can also be reported by patients (e.g., “I’ve used this tool and it's made me feel worse”). Feedback is always reviewed before it is sent back to the intervention developer, as service providers “*don't want to keep on sending stuff over without really thinking it through first”*. It is important that clinicians feel they work in partnership with the intervention developer company, and that their feedback and concerns are responded to quickly and subsequently implemented.

Routinely auditing iCBT data was stated by service providers to be important in improving how the service administers iCBT and may result in changes to service provision. Creating tools and reference documents aids supporters in their use of the intervention. For example, it was stated that administrative staff appreciated ‘Frequently-Asked-Questions’ guides to help answer questions for clients contacting the service. For clinical staff, general service guidelines, templates for online reviews, outline summaries of each iCBT program were developed. Like any other intervention within the service, supervision of digital service providers can make recommendations to supporters to switch clients to different interventions based on their needs (e.g., from bibliotherapy to iCBT, or vice versa). Line management within the service for iCBT is important in identifying and understanding barriers to iCBT usage, subsequently actioning on these, and evaluating supporters with regards to the minimum digital caseload requirement. This is operational goal that is actualized through personal development plans, which outline the division of work that a supporter should impose on their workload. Where supporters experience issues with using iCBT, the issue is addressed quickly through routine line management. Barriers identified during the appraisal of one supporter can create benefit for the wider team if appropriately addressed.

The importance of defining pathways when implementing iCBT was summarized succinctly by one participant: “*it's about having a really good understanding of what are the factors for recovery because if you’ve got a really good product and you put it into a lousy pathway, it can't deliver, which then gives it a bad reputation*.”. This process can also result in novel or innovative uses of the intervention within pathways for certain presentations (e.g., testing the use of iCBT as a prequel to face-to-face therapy) or increasing access (e.g., direct-to-iCBT pathways which allow patients access to iCBT without the need for formal triage).

### 3.3. Domain 3: patient experience of receiving iCBT as part of treatment in mental healthcare services

Patients generally reported positively on several platform features and some reported issues with the platform functionality. Regarding intervention content, patients appreciated that the program contained appropriate content and tools to address the problems the person is going through, and for patients who had received previous therapy (e.g., face-to-face CBT) reported that, in this instance, iCBT and its content was not redundant.

Participants stated that they had a positive assessment experience upon entering the service. Patients reported feeling well supported by their supporter to prepare for discharge from iCBT; patients were offered more supported iCBT sessions if they were not ready to be discharged, had their sessions tapered down in advance of discharge, were offered continued use of the intervention in an unsupported format and were offered further treatment (e.g., face-to-face therapy) if required. Participants stated that there were clear and defined procedures for cancelling or rescheduling treatment appointments. Some reported receiving helpful reminders from the service through email and text message.

Where patients received telephone calls to conduct their review sessions, they cited as helpful that supporters would provide typed summaries of the telephone call using the online support function through the iCBT platform. Patients stated that they appreciated when clinicians tailored content recommendations based on the problems presented by patients. Patients stressed the importance of telephone support in increasing adherence and normalizing their presenting problems. Patients also stated that more guidance was needed from the supporter and service regarding how to use the program and its tools. Supplementary Material S4 contains supporting quotes for each of the illustrated categories as part of this domain.

### 3.4. Domain 4: contextual considerations for implementing iCBT (findings combined from service provider and commercial iCBT representative groups)

Technology issues were cited as a barrier to iCBT implementation, for example, supporters may have to manually enter psychometric measures completed on the iCBT platform into electronic health records, meaning a duplication of effort. When mandates are issued at a national level within a health system that require services to change how they treat certain presentations (e.g., comorbid physical and mental health difficulties), services can lag behind in their adoption, subsequently causing confusion around the applicability of iCBT to these patient pathways. Training for new hires was raised, especially as “*…the training courses for working digitally, the PWP and the high intensity [courses] aren't really fit for purpose, they don't cover digital”.* This can result in newly qualified supporters having false expectations of working primarily in a face-to-face modality, when in reality the service employs iCBT to a high degree. Supporters can perceive the implementation of iCBT as a threat to their role, or a biased view of healthcare delivered only face-to-face, and that these attitudes are generally come from of a lack of previous exposure to or knowledge of these types of interventions. Cost increases associated with changes to the pricing model of iCBT can create issues around the perceived value of the intervention to customers, and may impact on contract renewals. Additional costs include, for instance, integration of iCBT with other software in the health system (e.g., electronic health records). Heterogeneity present within a healthcare market (e.g., private healthcare systems), places greater demand on commercial iCBT representatives during implementation however, markets with higher levels of homogeneity (e.g., publicly funded health systems) demand fewer resources when iCBT is implemented across different services.

COVID-19 has increased use of iCBT, where one service provider participant described this dramatic change as a complete “*paradigm shift”*. New services have adopted digital interventions and those more established have experimented with new ways, for example, delivering iCBT to individuals with severe depression/anxiety or borderline personality disorder, employee populations and services for children and young people. However, one service provider participant stated that it remains to be seen if iCBT is “*sticky after we go back to normal*”, but the attitudes of patients and clinical staff towards iCBT have changed for the positive due to COVID. Healthcare systems that recognize the potential of digital technologies, like the IAPT, is largely supportive of digital interventions by mandating it through their service design frameworks (e.g., incorporating iCBT as an option for the treatment of mild-moderate depression and anxiety). IAPT services all have a similar structure that allows for a scalable “*plug and play*” model for implementing iCBT. The perseverance of leadership to achieve reduced waiting times, greater access and maintain recovery rates, helps in creating a culture that maintains “*passion and dedication”* through problem solving and risk taking around iCBT to bring it to the point of patient benefit was highly important. These, alongside staff shortages and costs, are motivations for increasing the uptake of iCBT among staff within services. Stakeholders from both groups reported on how persevering with the use of iCBT over time can improve outcomes achieved through it. One participant in the service provider group estimated that it took 3–4 years for the service to realize the potential of iCBT fully.

## 4. Discussion

The current study qualitatively examined the experience of 3 groups; two stakeholder groups – commercial iCBT representatives who are employees of an iCBT intervention developer and service-providers – associated with the implementation of iCBT within services, and the patients receiving treatment. The findings identified across the three groups are certainly linked; the implementation effort is coordinated by commercial iCBT representatives and service providers, with patients ultimately experiencing the results of this effort. In discussing the results, we will link categories identified in each stakeholder group together in order to provide an interpretation of the meaning, relevance and how these findings relate to the wider literature base.

### 4.1. Leadership

Commercial iCBT representatives stated that engaging “the right people” at varying levels of the organizational hierarchy to drive the implementation was important from their point of view, and service providers emphasized the importance of leadership over several categories. Given its importance, we conceptualized leadership within our study as the ability of individuals within services to create and enact effective systems that support the use of the intervention and assist supporters in its utilization.

Within implementation science, leadership is a widely cited determinant that is posited to impact on implementation success (21–23), with Damschroder et al. (22) defining it as the level of “commitment, involvement and accountability of leaders and managers with the implementation”. Effective leaders allocate staff resource to support the implementation effort, obtain buy-in from other leaders within the health system, use evidence-based practice to inform implementation decisions, foster cultures that are conducive to change and learning and develop work structures (e.g., processes, procedures) for staff using the innovation (24–26).

However, it remains that the operationalization of leadership as a construct and understanding of its mechanisms that impact on outcome are still poorly understood, and there may be conceptual differences between the terms “leadership”, that is the ability to motivate, give feedback and support work, and “management”, which is the efforts conducted to actualize the vision of leadership (27, 28). Further considering this uncoupling of leadership and management, we observed in the current findings a large category that was associated with facilitating supporters in doing their work as part of iCBT (“Creating appropriate work structures for supporters in the delivery of digital”). Indeed, management has famously been defined as “the organ charged with making resources productive” (29); in the current instance, this can be seen to consist of providing supporters with the tools to make reviews easier, actioning their feedback, helping them contribute towards service goals and making sure the processes they work in (pathways) are set-up well. The findings of the current study provide support for the coupling of management and leadership together; digital champions and management work through various defined structures to actualize the wider goals of the service related to iCBT, illustrating how the two constructs are interlinked in the current study.

Further contributing to limitations associated with conceptualizing leadership, accounts of the effects of negative or “bad” leadership on the implementation of innovations in healthcare are few. Impacts of “bad” leadership include negative attitudes towards the leader and organization, employee deviance and lower levels of job satisfaction, job performance and commitment (30, 31). No negative experiences related to leadership were reported by service-based participants in the current study, nor have they been reported within iCBT implementation literature. For the current study, this could be a by-product of the service being well-versed in using iCBT as part of their delivery model, where reports of “bad” leadership, or reports of “what not to do” for leadership when implementing iCBT may be more salient in services where the implementation has either failed, or is just beginning. Relatedly, the retrospective nature of this study, where participants were requested to provide an account of a historic experience, may have resulted in participants omitting points related to ineffective leadership.

Digital champions, staff members who are given responsibility to promote the use of digital within the service, were implicated in several of the identified categories. This group of individuals can be most likened to opinion leaders, who are self-selected or nominated individuals within certain groups that act as role models for a specific behavior or activity (e.g., promoting iCBT use), are perceived positively by the group they originate from (e.g., bottom-up approaches) and are capable of exerting influence on the targeted behavior (e.g., increasing iCBT use among other psychological wellbeing practitioners within the service) (32–34). Digital champions were recognized by both commercial iCBT representatives and service providers as core to the implementation of iCBT as they motivate other staff, train others to use the intervention, work with staff who have problems and have deep knowledge of the intervention. In an analysis of the role of opinion leaders in healthcare change initiatives, it was cited that conflicts between opinion and expert leaders can cause difficulties when implementing change (34). However, in the current sample, digital champions acted as both opinion *and* expert leaders; initially, the service limited the role to digitally experienced staff but eventually opened it to everyone, where they acknowledged that all that was needed was an interest in digital working. Implementation science theory also recognizes the influence of opinion leaders. For example, Greenhalgh et al.'s (23) work on diffusion of innovations acknowledges the positive impact of harnessing opinion leaders when considering the implementation of an innovation, and subsequent studies investigating their influence in fields outside of healthcare have all cited their effectiveness (35–37).

### 4.2. Training

Training initiatives described within our dataset consisted of 1) the training of supporters in the use of iCBT and the development of online training materials by commercial iCBT representatives and 2), training initiatives for new starters and on-going training for new programs and procedures by service staff. The training protocol delivered at this unique service contains elements cited in several other trials. For example, training was performed to develop proficiency and comfort with iCBT (e.g., studies 38 & 39) and to allow for the development of specific competencies, like technical proficiency and writing progress reviews (13, 39, 40). Supporters were also are provided with extra training resources (e.g., an online help center), similar to what is reported by Hadjistavropoulos et al. (15).

Where iCBT can be considered an evidence based intervention (EBI), training clinical staff in EBIs is done to develop knowledge, technical skills and therapeutic competencies needed for intervention use and improve their attitudes and adherence to the EBI, but does not always achieve this intended result (41–44). Historically, training in the use of EBIs has largely consisted of a workshop and subsequent reading of a relevant intervention manual, which has been shown to be ineffective in regards to competency and skill acquisition (41, 42). A recent systematic review of 76 studies examining the impact of different training approaches on clinician-relevant training outcomes highlighted the superiority of intensive training, consisting of over 20 contact hours with two or more follow-up components, over all other approaches (43). As a caveat to this finding, the authors state that where intensive training is most successful, it can be highly time and cost intensive and thus should be employed tentatively until further research delineates the core components of what makes multi-component initiatives successful. Interestingly, and in response to this caveat, no participant in the service-based stakeholder group remarked negatively on iCBT training in terms of time or cost. This may be a product of the relatively positive view that this service has towards iCBT, and different experiences could be identified in services new to iCBT or having difficulties with its implementation. However, it remains that in a high-performing service, intensive training appears to become part of routine operations as opposed to a one-off, exceptional initiative.

When interpreting these findings, it can also be posited that the context where this training occurs (IAPT Services, England) may have acted as a facilitator. The clinician/supporter workforce implicated in this sample consisted of Psychological Wellbeing Practitioners (PWPs); psychology graduates that receive specialized postgraduate training in a range of low-intensity interventions (45). The structure of the iCBT program in use, SilverCloud, was originally built to reflect the work of PWPs, in that the main therapeutic component is delivered by the iCBT platform (similar to group therapy and bibliotherapy). However, iCBT has been delivered by a wide variety of professionals, including trained volunteers, registered mental health professionals and general practitioners [e.g., studies (46–49)], and it has been recognized that clinician-specific variables (e.g., clinical experience, attitudes towards iCBT) can impact on training requirements (41). Given the success of iCBT when administered by supporters of different backgrounds, certain authors have postulated that specific competencies are required by supporters administering iCBT [e.g., (50, 51)] and, more widely, telehealth (52–54). Where the existing competencies of supporters in the current study may have been facilitative of the uptake of iCBT, future implementation work should acknowledge that the training needs of professionals can vary across groups (e.g., charity volunteers vs. psychotherapists), settings (community vs. secondary care) and conditions (e.g., common mental disorders vs. long-term condition management).

### 4.3. Context

The domain of context was broken down into two sub-domains – contextual facilitators and contextual barriers. Regarding barriers, negative supporter attitudes towards iCBT reportedly arose through a combination of a lack of previous exposure to iCBT and expectancies as to how mental healthcare should be delivered (e.g., face-to-face). This finding is not novel, where previous papers report negative clinician attitudes relating to the effectiveness (55), quality and restrictive nature of iCBT impacting on the ability to generate a therapeutic alliance (13). It has also been reported elsewhere that negative attitudes towards iCBT can come from a previous lack of exposure to these interventions (38). A study that applied the non-adoption, abandonment and challenges to scale-up, spread and sustainability of healthcare technologies of Greenhalgh et al. (56) to gain insight into the implementation of an iCBT program for insomnia (iCBT-i) found that therapist attitudes improved across the observed implementation period (16). Indeed, it may be the case that increased exposure and familiarity with iCBT over the time may allow clinical staff charged with supporting iCBT to overcome prejudices and biases. For example, where our findings illustrate that the current service continuously disseminates examples of positive iCBT performance, this may encourage clinical staff to engage in a reappraisal of previously held thoughts about the limited effectiveness of iCBT. Given the systematic nature of the service towards implementing iCBT in the current example, it can be seen that any negative attitudes are addressed through leadership and training structures illustrated in Table 4, which further emphasizes the importance of the two aforementioned findings and illustrates how they combine to change attitudes around iCBT.

Facilitating factors associated with the IAPT model consisted of the support within the health system for the use of digital interventions, the creation of workforces that are habituative to digital implementations and the mandating of increased access targets, subsequently creating a need for digital products like iCBT. Indeed, there are a number of structures that support and advocate for eHealth initiatives within the United Kingdom. Firstly, the majority of NHS-operated services are based on guidelines developed by the National Institute for Health and Care Excellence (NICE), and the guidelines developed for the treatment of common mental health disorders advocate for the use of iCBT. Relatedly, NICE has developed a set of standards that digital health technologies can be compared against for services and commissioning groups to be able to identify what levels of evidence these interventions need to achieve (57, 58). Secondly, a collaboration between NHS England and England's department of health and social care resulted in NHSX, a national body responsible for setting the strategy for digital transformation within the country (59). It can be stated that, where service models or governmental agencies recognize the benefits of iCBT and eHealth initiatives, it strongly facilitates the use of these interventions (e.g., NHSX, E-Mental Health Strategy for Australia).

iCBT is currently only advocated for use in mild-moderate presentations of depression (60) within the NHS, but evidence is beginning to emerge for their use in more severe populations both within (61) and outside the NHS (62, 63). Given the reliance on NICE guidelines within the NHS, these can potentially limit services in experimenting with iCBT use cases that deviate from what is supported, which subsequently can hinder guideline amendment or improvement due to lack of innovation around use cases (61). Despite the presence and use of digital interventions such as iCBT across the NHS [e.g., SilverCloud used by 70% of IAPT services (64)], training courses for psychological wellbeing practitioners were stated to not incorporate this within their curriculum. Considering this, it may be inappropriate to place full responsibility on services to fully train PWPs in the use of digital interventions like iCBT at the outset, but rather it may be the responsibility of commercial iCBT representatives to foster and encourage the capacity to sustain a program of training in the long-term.

Both service providers and commercial iCBT representatives highlighted the facilitative nature of time, where it was cited that service and staff procedures around iCBT can evolve for the better when sufficient effort is sustained throughout the time period. Process models of implementation, which define a set of steps (or processes) that need to be undertaken to arrive at implementation success), have a similarly implied temporal element (65). One of these models, the Exploration, Preparation, Implementation and Sustainment framework [EPIS (66);], states that throughout an implementation effort, services proceed through each of these 4 phases in a linear fashion, and the current findings are illustrative of this. For example, supporters stated that the invite script used to introduce patients was refined over time to improve it. However, before arriving at this point it can be implied that the service acknowledged the need for an invite script through an exploration phase, developed it in a preparation phase, evaluated it in the implementation phase, and it was further refined through the sustainment phase to increase its efficiency. Relatedly, the Dynamic Sustainability Framework (67) postulates that innovations are not optimized when initially implemented, and improvements in innovation delivery occur due to attempts to ‘fit’ it to the needs of a given setting over time.

### 4.4. iCBT intervention developers and commercial iCBT representatives

The current study highlighted the role of commercial iCBT intervention developers and their employees in implementing iCBT within healthcare systems. Although these reports consist of employees of only one such intervention developer, SilverCloud Health, it provides insight into the experience of a group that is relatively undocumented within the literature base. The findings highlight that commercial iCBT representatives contribute their efforts towards building the required team structure to best support their customers in regards to training, problem-solving and disseminating best-practice use cases of iCBT. In this regard, commercial iCBT representatives can be most likened to Implementation Support Practitioners [ImpSPs (68–70);]. In defining the role of ImpSPs, Albers, Metz & Burke (69) state that they are individuals who work with staff that are required to enact or implement a specific change, to the extent that what is being implemented becomes sustainable and scalable. Metz et al. (70) state that the competencies of ImpSPs fall under 3 domains; co-creation and engagement (e.g., engaging the relevant stakeholders in the implementation process to design appropriate pathways for iCBT), ongoing improvement (e.g., imbuing values around learning, feedback and evaluation as part of service delivery) and sustaining change (e.g., creating relationships, teams and digital champions that ensure the sustainability of iCBT). In an example specific to iCBT literature, Hadjistavropoulous et al. (15) stated that the presence of an ‘external facilitation unit’ akin to ImpSPs, that managed an iCBT website, educated clinicians, provided technical assistance and sourced funding for iCBT, was perceived by participants to be a facilitator of implementation. Given the presence of commercial intervention developers within the healthcare field, it may be important to further build upon the competencies of ImpSPs [as illustrated by Metz et al. (70)] when training new employees within the intervention developer company. However, collaboration between public and commercial sectors can be difficult with regard to competing interests (71, 72) and therefore services should rely on evidence standards available in their countries (e.g., IAPT Assessment Briefs or NICE standards) to make judgements on which commercial intervention developers to engage with.

### 4.5. Patient experience

Commensurate with the established literature (18, 55, 73), patients reported a positive experience of iCBT as delivered by the service. Therefore, it may be appropriate to posit that an effective implementation results in positive experiences for patients. Therapist support in particular is instrumental in maintaining adherence, which is reflective of the literature base for iCBT (74–76). However, the difference between online (e.g., internet-facilitated asynchronous, text-based communication) and telephone supported iCBT is less established. For example, and in regard to clinical symptoms, Lindner et al. (77) found significant decreases in symptoms in patients after a course of iCBT, but no difference was found between the telephone and e-mail supported groups. In a comparison between groups consisting of telephone support vs. online support, Pihlaja et al. (78) found higher levels of adherence and greater reductions in depressive symptomatology in patients in the telephone support group. Although not definitive, a tentative link can be drawn between these two studies and the role of the work of the PWP supporter in IAPT, where the intervention (e.g., iCBT, bibliotherapy) conveys the main therapeutic principles and the supporter may contribute towards increasing adherence through processes of supportive accountability when a telephone session is scheduled (79).

A positive patient experience of the treatment journey from assessment, to treatment, to discharge, were all noted by participating patients and in particular, it was noted that they experienced a feeling of making a collaborative, positive decision when selecting iCBT as their treatment of choice. The role of the supporter in creating positive expectations of treatment at point of assessment draws parallels to the work of Jardine et al. (19), who conducted a study in a similar IAPT care context and identified similarly that expectations of iCBT are high and patients experience it positively from the outset. This collaboration between client and supporter can be beneficial in overcoming iCBT-related barriers, where it has been illustrated elsewhere that human support can mitigate against intervention-related issues, such as technology problems, which may otherwise cause patients to abandon the intervention (80). These findings reflect and reinforce previous literature surrounding the supporter implicated in iCBT; where the intervention communicates the active treatment ingredients, the supporter creates positive attitudes towards iCBT through a positive assessment and treatment allocation experience, adherence through processes of supportive accountability and retention through facilitating discussion around issues encountered. However, from an implementation perspective, our findings suggest that incorporating routine telephone support into iCBT enhances patient experience and facilitates their engagement.

## 5. Strengths and limitations

This study reports on the experience of participants within a service that has successfully implemented and scaled iCBT. Therefore, this research contributes to a small, but growing field of literature that seeks to understand the experience of relevant stakeholders who participate in the implementation of iCBT [similar to Folker et al. (13); Hadjistavropoulous et al. (15)]. A further, related strength of the research is its inclusion of a diverse group of stakeholders, including commercial iCBT representatives working for an iCBT intervention developer company and patients. The closest example of a group occupying a role similar to that of the commercial iCBT representatives in the current study is that of Hadjistavropoulous et al. (15), who illustrate that an ‘External Facilitation Unit’, a publicly funded group that was perceived positively due to it assisting clinic staff with the management of iCBT.

A limitation of this study is that is that participants within the service-based stakeholder group come from a single, high-performing service within the England's NHS. Therefore, the findings generated are limited to the perspective of this group. Also for patients, those that participated in the study all reported positive perceptions of their treatment and experience with the service and also only consisted of those who had 4 + sessions of supported iCBT. Those who completed fewer sessions or were marked as a treatment dropout may report differing experiences. Similarly, this study captures the experience of only one commercial iCBT company in a market. Lastly, although the findings appear transferable their nature begs caution in terms of generalizability for two reasons; one, the small sample size across each of the three groups, and that findings may be more pertinent for similarly high performing and successful services. Future research should focus on replicating a similar inquiry with different stakeholder groups within similar population (e.g., other services, patient groups, commercial iCBT representatives).

## 6. Conclusion

The current work helps to contribute to the literature in understanding how iCBT is implemented and operates in a dynamic healthcare context. The inclusion of a diverse group of professionals and roles as part of the three main stakeholder groups has resulted in a robust review of the experience of implementation and contextual considerations for implementing iCBT. The most salient factors have been discussed and include leadership, training, understanding context, the role of stakeholders, and the patient experience; each seem to be related to highly successful implementations. This current work can be further used to help services and researchers in implementing iCBT in routine care settings. Although the focus of implementation science is primarily on improving the use of innovations within services (11), the current findings provide support for the idea that a well-implemented iCBT initiative can create an overall positive experience for patients using it.

## Data Availability

The datasets presented in this article are not readily available. However, the qualitative transcripts can be made available, in anonymized format, on request from the corresponding author for the purposes of further research within a related field of study. Please note that, due to the qualitative nature of the data, sections of text will be redacted to protect the confidentiality of participants. Requests to access the datasets should be directed to DD - daniel.duffy@amwell.com.
