# Supplementary material for: Implementing internet-delivered cognitive behavioral therapy in healthcare services: a qualitative exploration of stakeholder experience
Source: Front Digit Health. 2023 Sep 26;5:1139125. doi: 10.3389/fdgth.2023.1139125 (PMC10562631; doi:10.3389/fdgth.2023.1139125)
Supplement: Supplementary file 1 [file Datasheet1.zip › Data Sheet 1_v1/Table 1.DOCX]

**1)** Can you tell me about your role? How do you currently work or how you have worked in the implementation of the SilverCloud solution? How many years have you been in this role for?

**2)** Explain the following to the participant:

*“When implementing SilverCloud in healthcare services, different types of personnel from parts of an organisation become involved at different levels.*

*To explore this a little bit more, I’d like for you to go back and tell me about your experiences of implementing SilverCloud.”*

**A:** Firstly, working as a member of the **customer success team**, I understand that you may have been involved in a number of different aspects of the implementation such as…

1. Working with the relevant teams to ensure the account is ready to go-live in terms of technical integration
2. Identifying and working with Intervention/Digital Champions at sites
3. Training
4. Facilitating growth within the account (e.g. identifying new user population)
5. Working closely with sales to facilitate account renewals and upsell

**A:** Firstly, working in a **product/development role**, I understand that you may have been involved in a number of different aspects of the implementation such as…

1. Working with customer success in regards to technical integration and facilitating growth within certain accounts
2. Alerting to customers any changes in the user interface or technical improvements within the platform.
3. Providing technical support
4. Working with customers to improve the platform

**A:** Firstly, working in a **sales/commercial role**, I understand that you may have been involved in a number of different aspects of the implementation such as…

1. Identifying potential new customers
2. The procurement of the intervention
3. Working with services to introduce SilverCloud
4. Working with the customer success team to manage the account, renewals and upsell

Have I gotten this right? Is there anything I may have missed about your involvement in the implementation of SilverCloud?

**B:** Can you tell me about your experience of each of these, starting with *area*?

**C:** Based on everything you’ve just told me, I would like to elicit your feedback

- What works well?
- What doesn’t work well?
  - Follow up: What could be improved upon?
- If participant struggles, bring back the strategies to their experience
  - *“what have you done in the past that you think was effective or ineffective?*
  - *“What have others done that you thought was effective or ineffective?”*

**3)** Based on your experience and what we have talked about, do you believe that contextual factors impacted on the implementation of SilverCloud?

Prompts

- context (inner, outer, political, cultural factors, commercial, competitive)
- Provide examples, where necessary (e.g. the need to meet treatment targets around certain groups as outer/political context, leadership issues as internal context)
- Rephrase the question: “if they impacted or ***can*** impact on the implementation...
  - Bringing the question to a general level, then back to specifics can help focus the participant.

**4)** From your experience, what, in your view, are the aspects of the implementation process or influencing factors that matter most?
