# Supplementary material for: Implementing internet-delivered cognitive behavioral therapy in healthcare services: a qualitative exploration of stakeholder experience
Source: Front Digit Health. 2023 Sep 26;5:1139125. doi: 10.3389/fdgth.2023.1139125 (PMC10562631; doi:10.3389/fdgth.2023.1139125)
Supplement: Supplementary file 1 [file Datasheet1.zip › Data Sheet 1_v1/Table 2.DOCX]

**1)** Demographics – gather participant information on age + gender.

**2)** Explain the following to the participant:

*“When SilverCloud is implemented in healthcare services, service users can be introduced to and interact with the online programme in a number of ways.*

*To explore this, I’d like to ask you some questions about your experiences of using SilverCloud”*

**A:** As a service user of an Improving Access to Psychological Therapies service, I understand that you may have encountered the following before, during and after using the SilverCloud Intervention…

1. Being referred by a GP or self-referring to an IAPT service for psychological services
2. Being assessed by a PWP for service eligibility
3. Using the online intervention throughout the treatment period
4. Receiving online or telephone support from your PWP
5. Contacting the service where you had any issues or queries, such as missing or rescheduling an appointment, pausing treatment due to a holiday, etc.
6. Being discharged from the service

Have I gotten this right? Is there anything I may have missed about your experience of attending IAPT Services?

B: Can you tell me about your experience of each of these, starting with *x* (areas listed above)?

C: Based on everything you’ve just told me, I would like to elicit your feedback about the service you were provided

- What has worked well in the service you were offered?
- What didn’t work well in the service you were offered?
- What could work better in how the service was delivered to you?

**3)** Based on your experience and what we have talked about, do you believe that contextual factors impacted on your treatment experience of SilverCloud?

Prompts

- context (inner, outer, political, cultural, factors, commercial, competitive)
- Provide examples, where necessary

**4)** Overall, were you satisfied with the treatment you received?

- What was central to this?
