# Supplementary material for: Implementing internet-delivered cognitive behavioral therapy in healthcare services: a qualitative exploration of stakeholder experience
Source: Front Digit Health. 2023 Sep 26;5:1139125. doi: 10.3389/fdgth.2023.1139125 (PMC10562631; doi:10.3389/fdgth.2023.1139125)
Supplement: Supplementary file 1 [file Datasheet1.zip › Data Sheet 1_v1/Table 3.DOCX]

**1)** Can you tell me about your role? How do you currently work or how you have worked in the implementation of the SilverCloud solution? How many years have you been in this role for?

**2)** Explain the following to the participant:

*“When implementing SilverCloud in healthcare services, different types of personnel from parts of an organisation become involved at different levels.*

*To explore this a little bit more, I’d like for you to go back and tell me about your experiences of using SilverCloud at the start.”*

**A:** Firstly, working in a **management/directorial role**, I understand that you may have been involved in a number of different aspects of the implementation such as…

1. The procurement of the intervention
2. Designing pathways and procedures around the intervention
3. Training
4. Monitoring and evaluating the progress and outcomes of the intervention

**A:** Firstly, working as a **Service Manager/Team Manager/ Digital Lead**, I understand that you may have been involved in a number of different aspects of the implementation such as…

1. Training
2. Creating and actioning service procedures around the digital intervention
3. Facilitating the work and learning of others around the intervention
4. Identifying problem areas around the implementation of the intervention and addressing these.

**A:** Firstly, working as a **PWP**, I understand that you may have been involved in a number of different aspects of the implementation such as…

1. Training (both SilverCloud and in-service)
2. Assessing suitability for an online intervention
3. Bringing patients on a digital therapy to supervision
4. Following up and managing patients on a digital intervention

Have I gotten this right? Is there anything I may have missed about your involvement in the implementation of SilverCloud?

**B:** Can you tell me about your experience of each of these, starting with *area*?

**C:** Based on everything you’ve just told me, I would like to elicit your feedback

- What works well in terms of implementing?
- What doesn’t work well in terms of implementing?
  - Follow up: What could be improved upon?

**3)** Based on your experience and what we have talked about, do you believe that contextual factors impacted on the implementation of SilverCloud?

Prompts

- context (inner, outer, political, cultural factors, commercial, competitive)
- Provide examples, where necessary (e.g. the need to meet treatment targets around certain groups as outer/political context, leadership issues as internal context)
- Rephrase the question: “if they impacted or ***can*** impact on the implementation...
  - Bringing the question to a general level, then back to specifics can help focus the participant.

**4)** From your experience, what, in your view, are the aspects of the implementation process or influencing factors that matter most?
