# Supplementary material for: Implementing internet-delivered cognitive behavioral therapy in healthcare services: a qualitative exploration of stakeholder experience
Source: Front Digit Health. 2023 Sep 26;5:1139125. doi: 10.3389/fdgth.2023.1139125 (PMC10562631; doi:10.3389/fdgth.2023.1139125)
Supplement: Supplementary file 1 [file Datasheet1.zip › Data Sheet 1_v1/Table 4.DOCX]

## Supplementary Table - Sub-domains, categories and quotes associated with Domain 3 – Patient experience of an iCBT treatment pathway, based on data from the patient group (N=7)

| Sub-Domain | Category | N | Quote |
| --- | --- | --- | --- |
| Patient experience of the iCBT platform | Patients state the flexibility and accessibility of the platform as positive aspects of iCBT | 6 | I have 2 small children and it can be incredibly hectic and well—hectic. So, actually, something that I could pick up in the moments where I did have time, was really good because nobody was checking when I was doing anything, it was just that I could make my way through it at my own pace. |
|  | Patients appreciated that the program contained appropriate content and tools to address the problems the person is going through | 5 | It was very much kind of like I was in a position where I was trying to distance myself from my feelings. It [the programme] was forcing me to address it, which is better overall, I would say. |
|  | Patients appreciated how the platform enabled them to take and use the content they needed, while filtering out content that was less relevant | 4 | Certain modules won’t work for me, I could never get into the slow breathing and all that stuff…. There were some bits, the visualisation parts of it, yes, that was more for me so, it’s almost trying to help tailor the programme early on, I think. |
|  | Patients expressed a need for more guidance within the intervention regarding how to effectively use it | 4 | When I started playing around with it a bit more, I would go, oh, there are settings, oh, there is this— so, it’s almost like one of these things you don’t actually—it’s like one of these things when you’re using these things is actually to have it upfront, rather than hidden away |
|  | Issues with platform functionality, including tool layouts, presentation of questionnaires, length of mindfulness exercises and security features (i.e. requiring repeated logins) | 4 | when it gives you the questionnaires to follow, and you get to the last question...all of a sudden, it shoots to the bottom of the screen and it’s saying, you haven’t answered these questions and you have to scroll back up to find the questions you haven’t answered. It does some odd behaviour sometimes. |
|  | The integrated reminder function on the platform is helpful and useful in structuring patient iCBT usage | 3 | Yes, there are some good bits to it, and I think the bit I hadn’t realised, is that you could actually set up a notification to say, please log into your programme. |
|  | Patients who had received previous therapy (e.g. face-to-face CBT) reported that iCBT and its content was not redundant | 3 | I would say it was repeating what I had heard before, but then the tools I could use were different to what I had been given before...things like worry trees, and having the relaxation videos on there...the content was quite similar to therapy I had before, but then, how to work on it, was different and definitely more advanced. |
|  | Patients appreciate being able to download and print content for instances with no internet connection | 2 | My daughter is doing the home-schooling, and she has taken over my room with my computer, which is really irritating... So, I don’t get much time to catch up with what I’m supposed to be doing. So what I did do was, I actually printed off a lot of the stuff from the online course, and I go through those regularly, and the notes that the therapist gave me. So, I actually have it all to hand, to read. |
|  | Patients reported the platform to be an aesthetically pleasing experience | 2 | Presentation is very good, the mixture between the actual text and the drawings. Sometimes real people—talking; at the beginning, you get two people talking about the next unit and then you get people talking about their own experiences, that is very helpful |
| Patient experiences of the administration of treatment by the service | Positive assessment experience; supporters collaborated with patients to decide on iCBT and normalised their treatment-seeking. | 6 | It [the assessment] was all very clear...it was set out to me exactly what was going to happen and that kind of structure—it gave me a bit of comfort that okay, I’ve got this step, and then this step...you know what is going to happen in the next couple of weeks, for example. |
|  | Feeling supported by supporter to prepare for discharge from iCBT. | 5 | Originally, we spoke about being discharged in my second to last session, so, with that, we increased the timescale...we went to having monthly chats and that allowed me to be able to confirm that I was definitely ready for that; it was able to sit there—when she called and she was able to say that she was still there, for when I needed her, that it wasn’t a problem |
|  | Clear and defined procedures for cancelling or rescheduling treatment appointments | 3 | I remember it being quite easy...there is a general number that you can call, and then he got in touch with me to rearrange another meeting. And I know that there is a certain time limit that you can’t—obviously, people don’t want their sessions to be cancelled at the last minute. I don’t think mine was last minute, but it could have been within 24 hours, so, it was quite good that I didn’t suffer any penalties or anything because something cropped up. |
|  | Multiple reminders (text message and e-mail) sent by the service helped to maintain engagement in treatment | 2 | I would say that I think they did a very good job of reminding me because I received an email and a text, both letting me know when my appointment was, and it was also available on the app. |
| Patient experiences of their clinical supporter | Patients found typed summaries of telephone calls using the online support function helpful in structuring their future use of the program | 5 | We would finish and then I would get a notification saying I’ve got a message, and then, yes, it would be a summary, and then next steps—what to do. |
|  | Patients reported that more guidance is needed from the service regarding how to use the program and its tools | 4 | I’m not sure if I was told, but I didn’t realise that I would have access to SilverCloud post the 6 weeks...so I got a bit panicky and started trying to take photos of the whole thing. And then it was only when I got to my last session, [therapist] was like, “You’ve got access to it for a year.” And I was like, “Oh, okay.” So, I went through and deleted all of the hundreds of photos I had taken. |
|  | Patients appreciated when supporters tailored content recommendations based on their presenting problems | 3 | you have regular meetings with the therapist, that is over the phone, and then you discuss any issues that you have, and he will help you; maybe sometimes setting you certain tasks [on the iCBT programme] that he thought would be good for me. |
|  | Patients stress the importance of telephone supporter support in increasing adherence and normalising presenting problems | 2 | I do enjoy human interaction, even if it is just on the phone and I feel like if it if was typed online, for me, that wouldn’t—I would probably—not ignore it but I feel like I wouldn’t connect to it as well. |
|  | Patients reported that the initial awkwardness of telephone supported was alleviated by the supporter’s skill | 1 | At the beginning, I absolutely hated it... I felt awkward but that was more a personal thing and having to get over that was one of the biggest hurdles. Actually, she made me feel very comfortable, she put the confidence in me that she knew what she was talking about. If I wanted to sit there and rant, she would let me. If I needed a bit more pointing in the right direction, she was there to be able to do that and I can’t thank her enough, to be honest |
| Patient experience of the service referral process | Patients reported positive experiences of the online, self-referral process | 3 | I found the website easy to access and navigate through. I would say it was a positive experience overall with it |
|  | Patients reported speaking with GPs regarding mental healthcare as an easy and positive experience | 3 | I found it was actually a good experience. I got referred from the GP to self-refer...I thought she was lovely when she did that, she was very reassuring. |
|  | Patients report a preference for online referral over healthcare provider referral when they have previous negative experiences with treatment seeking | 1 | I was very put off from—I’ve since gone back to a GP—I’ve changed GP and gone back, but at that time, it was very much, oh, I’d really rather not go through that GP procedure because of previous experiences. |
